# Living Lab Data of Patient Needs and Expectations for eHealth-Based Cardiac Rehabilitation in Germany and Spain From the TIMELY Study: Cross-Sectional Analysis

**DOI:** 10.2196/53991

**Published:** 2024-02-22

**Authors:** Boris Schmitz, Svenja Wirtz, Manuela Sestayo-Fernández, Hendrik Schäfer, Emma R Douma, Marta Alonso Vazquez, Violeta González-Salvado, Mirela Habibovic, Dimitris Gatsios, Willem Johan Kop, Carlos Peña-Gil, Frank Mooren

**Affiliations:** 1 Department of Rehabilitation Sciences Faculty of Health University of Witten/Herdecke Witten Germany; 2 Center for Medical Rehabilitation DRV Clinic Königsfeld Ennepetal Germany; 3 Health Research Institute of Santiago de Compostela Santiago de Compostela Spain; 4 Center of Research on Psychological Disorders and Somatic Diseases Tilburg University Tilburg Netherlands; 5 Cardiology and Coronary Care Department IDIS, CIBER CV University Hospital of Santiago de Compostela Santiago de Compostela Spain; 6 Capemed Ioannina Greece

**Keywords:** eHealth, coronary artery disease, rehabilitation, mHealth, mobile health, mobile phone, app, apps, applications, personalized medicine, patient empowerment, living lab, coronary, cardiac, cardiology, heart, telehealth, telemedicine, monitoring, survey, surveys, experience, experiences, attitude, attitudes, opinion, perception, perceptions, perspective, perspectives, acceptance, technology use, usage

## Abstract

**Background:**

The use of eHealth technology in cardiac rehabilitation (CR) is a promising approach to enhance patient outcomes since adherence to healthy lifestyles and risk factor management during phase III CR maintenance is often poorly supported. However, patients’ needs and expectations have not been extensively analyzed to inform the design of such eHealth solutions.

**Objective:**

The goal of this study was to provide a detailed patient perspective on the most important functionalities to include in an eHealth solution to assist them in phase III CR maintenance.

**Methods:**

A guided survey as part of a Living Lab approach was conducted in Germany (n=49) and Spain (n=30) involving women (16/79, 20%) and men (63/79, 80%) with coronary artery disease (mean age 57 years, SD 9 years) participating in a structured center-based CR program. The survey covered patients’ perceived importance of different CR components in general, current usage of technology/technical devices, and helpfulness of the potential features of eHealth in CR. Questionnaires were used to identify personality traits (psychological flexibility, optimism/pessimism, positive/negative affect), potentially predisposing patients to acceptance of an app/monitoring devices.

**Results:**

All the patients in this study owned a smartphone, while 30%-40% used smartwatches and fitness trackers. Patients expressed the need for an eHealth platform that is user-friendly, personalized, and easily accessible, and 71% (56/79) of the patients believed that technology could help them to maintain health goals after CR. Among the offered components, support for regular physical exercise, including updated schedules and progress documentation, was rated the highest. In addition, patients rated the availability of information on diagnosis, current medication, test results, and risk scores as (very) useful. Of note, for each item, except smoking cessation, 35%-50% of the patients indicated a high need for support to achieve their long-term health goals, suggesting the need for individualized care. No major differences were detected between Spanish and German patients (all *P*>.05) and only younger age (*P*=.03) but not sex, education level, or personality traits (all *P*>.05) were associated with the acceptance of eHealth components.

**Conclusions:**

The patient perspectives collected in this study indicate high acceptance of personalized user-friendly eHealth platforms with remote monitoring to improve adherence to healthy lifestyles among patients with coronary artery disease during phase III CR maintenance. The identified patient needs comprise support in physical exercise, including regular updates on personalized training recommendations. Availability of diagnoses, laboratory results, and medications, as part of a mobile electronic health record were also rated as very useful.

**Trial Registration:**

ClinicalTrials.gov NCT05461729; https://clinicaltrials.gov/study/NCT05461729

## Introduction

The application of eHealth technology in cardiac rehabilitation (CR) is being increasingly adopted to enhance patient outcomes. eHealth, which involves the use of digital health technologies, has the potential to facilitate CR programs to offer better, more efficient, and cost-effective care. CR is a crucial aspect of the recovery process after a cardiac event, aiming to reduce the risk of future events and improve the quality of life of patients [1,2]. The European Society of Cardiology defines CR as a multifactorial intervention with core components in patient assessment, physical activity, diet/nutritional counselling, risk factor control, patient education, psychosocial management, vocational advice, and lifestyle behavior change, including patients’ adherence and self-management [3]. The CR process is typically divided into 3 stages. During phase I, patients discuss their cardiovascular risk factors and health situation in the acute clinic after a coronary intervention or surgery with their treating physician or a CR nurse. This brief phase lasts only a few days and aims to get patients moving as soon as possible, encouraging mild levels of physical activity [4]. Phase II, the reconditioning phase, occurs at inpatient or outpatient CR centers or even in the home environment with various levels of support. This multidisciplinary phase includes education on risk factors, supervised exercise training, and psychological support, with the goal of improving patients’ exercise capacity, functional mobility, and self-management skills [5]. In phase III, also referred to as the maintenance phase, patients continue their care in a community or home-based setting. Phase III is the longest and least structured phase of CR, aiming at lifelong self-care with continuous risk factor management and regular physical activity to maintain the achievements made during phase II [4,6]. However, adherence to a healthy lifestyle, including regular physical activity and risk factor management, during phase III maintenance is challenging and often poorly supported [7,8]. The main reasons for suboptimal adherence to phase III CR include patient-related factors (eg, motivation) and unsustainable costs for lifelong patient support in addition to usual care by general practitioners or cardiologists [9,10]. In addition, patient barriers such as time and travel burden may add to lower adherence and uptake of maintenance programs.

Information and communication technology in the form of eHealth applications has undergone recent developments by targeting reduction of possible barriers of initiation and continued engagement in CR [11]. The advantages of eHealth include less time investment and constraints due to the absence of travel, option of continuous monitoring, and possibility for patients to manage their disease independently [12,13]. The use of eHealth technologies allows for personalization and tailoring of CR programs to individual needs, leading to higher effectiveness and improved outcomes for patients. Furthermore, eHealth applications allow for different CR aspects to be targeted independently or in a combined and synergistic manner and may have positive effects on physical activity, medication adherence, mood states, anxiety, and depression in cardiac patients [14]. However, there is no uniform eHealth platform available combining all aspects of CR for patients with cardiovascular disease over the continuum of care, including phase III maintenance. Although challenging on a technological level, user acceptance and applicability in day-to-day setting are key for implementation and success of such a solution. In addition, factors such as technological skills, trustworthiness, and overall individual attitude toward eHealth need to be considered [15-17].

Based on this background, the goal of this study was to provide a detailed description of the patient perspective on the most important aspects to be included in an eHealth solution to assist phase III CR maintenance. This report is part of the multistakeholder project TIMELY, which aims at developing a personalized eHealth platform to assist patients over the continuum of the disease according to recent coronary artery disease (CAD) guidelines [18]. TIMELY employs artificial intelligence–powered CR components in a patient app connected with a patient management platform and decision support tools for case managers and clinicians. Additionally, artificial intelligence–powered conversational agents (chatbots) will be provided to engage in motivational conversations with patients based on behavior change techniques with the goal of optimizing program and exercise adherence. The development of the TIMELY eHealth solution is guided by a Living Lab approach that allows researchers to co-design innovations such as TIMELY with patients in a real-life context to increase acceptance [19]. Multiple feedback loops are included at pivotal developing stages, incorporating patients and clinicians in a modified Delphi approach [20,21]. Within the TIMELY prospective study, patients are equipped with different devices as part of the envisioned solution, including a long-term 3-channel electrocardiogram (ECG) patch, a hemodynamic monitor for blood pressure measurement and pulse wave analysis, and a wrist-worn activity tracker. This report describes patients’ needs and expectations for eHealth-based CR collected within the TIMELY Living Lab in CR centers from Germany and Spain.

## Methods

### Approach and Participants

To characterize patients’ needs and expectations for an eHealth-based phase III CR maintenance system, a guided survey was conducted at medical rehabilitation centers Clinic Königsfeld, Germany, and University Hospital of Santiago de Compostela, Spain, between July 2021 and March 2022, aiming at a representative sample of ~80 participants. Patients were asked to participate during their inpatient (Germany) or outpatient (Spain) CR program, and participants were recruited consecutively without further selection. Patients diagnosed with CAD were eligible while participating in a structured center-based CR program.

### Ethics Approval

This study complied with the Helsinki Declaration “Ethical Principles for Medical Research Involving Human Subjects” and was approved by the ethics committee of University Witten/Herdecke (115/2020) and Servizo Galego de Saúde (2021/190). All participants gave their written informed consent before participating in this study. This study is part of the TIMELY observational trial (ClinicalTrials.gov: NCT05461729), which aims to characterize the progress of patients with CAD during phase II and phase III CR.

### Patients’ Characteristics

Patients’ anthropometric and clinical data, including severity of CAD, type of intervention, and comorbidities (rated using the D’Hoore comorbidity index [22]) were extracted from electronic health records by clinical personnel. Patients’ highest level of education was documented and specified by country. Hauptschule and Educación primaria were defined as primary, Realschule and Educación secundaria obligatoria or vocational training as secondary, and Abitur or Bachillerato as tertiary education in Germany (DE) and Spain (ES), respectively. A university degree was classified as the highest educational category. For comparability and due to differing educational systems in Germany and Spain, the level of education was categorized as “lower/equal to high school” (first two levels) or “higher than high school” (all other higher levels).

### Interview-Based Survey

This survey was developed with experts from a clinical and theoretical perspective by using the Delphi method until consensus was reached. The survey (20 items) was composed of 3 parts: (1) importance of different CR components in general, (2) digital literacy and current usage of technology/technical devices, and (3) helpfulness of the potential features of eHealth in CR (Multimedia Appendix 1). Closed questions were used with a list of provided answers rated on a 5-point Likert scale (1=unimportant/not useful; 5=very important/very useful). A filter question was used, which optionally exempted participants who indicated that they would never use an eHealth platform linked to devices. These participants were asked for their reasons for refusing to use an eHealth platform. The survey was pretested with selected patients in Clinic Königsfeld, and adaptations for wordings were made, where necessary. The final version of the survey was translated to German (SW and BS) and Spanish (MSF and MA) by at least 2 researchers for each translation. The survey was conducted by researchers of the local rehabilitation center. Questions were read to the patients, and further explanation was provided if needed. Investigators documented the answers by using a paper-pencil version or an electronic version of the survey (Multimedia Appendix 1).

### Questionnaires

In a subset of 40 German patients with CAD, questionnaires were used to identify personal traits potentially predisposing patients for acceptance of an app or monitoring devices to document the progress of CR (ie, questions Q12 and Q13 of the survey). Psychological flexibility was assessed using the Acceptance and Action Questionnaire version 2 (AAQ-2) [23], and the Revised Life Orientation Test (LOT-R) [24] was used to identify patients’ optimism/pessimism. The Type D scale for social inhibition (DS-14) [25] was used to assess negative affectivity, social inhibition, and type D personality. In addition, the Positive and Negative Affect Schedule (PANAS) was applied [26].

### Statistical Analysis

Statistical analyses were performed using the open access program Jamovi (version 2.2.2, The Jamovi project) and SPSS (version 29, IBM Corp). Data are presented as mean and standard deviation, median and range for the Likert rating scales, or n (%) as indicated. Normality was tested using Shapiro-Wilks test. Between group differences were tested using independent 2-sided *t*-test or analysis of variance. Nonparametric tests were used to investigate group differences in Likert scale data (Mann Whitney *U* and Kruskal Wallis test). The associations of sex, age, education level as well as different psychological constructs with openness to using eHealth were analyzed between groups (general willingness [yes/maybe] and patients not willing to use eHealth [no]) by using chi-square test or Mann Whitney *U* test as indicated. To analyze the combined predictive values of multiple patients’ characteristics on eHealth acceptance, we used multivariate linear regression and naïve Bayes classification. The statistical significance level was set at *P*<.05.

## Results

### Patients’ Characteristics

Seventy-nine patients participated in the guided survey (Germany, n=49; Spain, n=30; 16/79, 20% females). The mean age (in years) of the patients was 57 (SD 7; range 37-79) (Table 1). In Germany, our sample population was comparable in terms of sex and age to patients with CAD in general (registry data) [27] and to patients with CAD undergoing CR in particular (mean 54.9, SD 7.0 years, in-house data). Further comparison of the study sample to German patients with CAD undergoing CR showed considerable similarity also in terms of ST-elevation myocardial infarction/non–ST-elevation myocardial infarction (~75%), number of affected vessels (1 vessel disease, ~30%-40%), and performed intervention (bypass, ~20%; all in-house data). For Spain, our study sample was comparable to patients with CAD undergoing CR in terms of age (~61 years), ST-elevation myocardial infarction/non–ST-elevation myocardial infarction (~85%), number of affected vessels (1 vessel disease, ~60%), and performed intervention (bypass, ~5%; all in-house data, region Galicia). Overall, in terms of the education level, 87% (69/79) of the participants were ≤high school and 13% (10/79) were >high school (Table 1). Comparisons between countries suggested good comparability even though the age (in years) of the Spanish participants (mean 62, SD 10) was higher than that of the German participants (mean 56, SD 6; *P*<.001), which was associated with a significantly higher burden of comorbidities (median ES 2.3, IQR 1-8; median DE 1.6, IQR 0-7; *P*=.03). The percentage of former smokers among patients with CAD in Germany was significantly higher than that in Spain (27/49, 55% vs 7/30, 24%; *P*<.001). Overall, 30% (24/79) of the included participants were active smokers. Of the 79 participants, >85% (67/79) indicated that they (highly) appreciated being involved in the planning of a future eHealth solution.

**Table 1:** Characteristics of the patients.

		Overall (N=79)	Germany (n=49)	Spain (n=30)	*P* value^a^	
Age (years), mean (SD)		57 (9)	56 (6)	62 (10)	.001	
**Sex, n (%)**						>.99
	Female	16 (20)	10 (20)	6 (20)		
	Male	63 (80)	39 (80)	24 (80)		
Height (cm), mean (SD)		172.3 (10.0)	175.9 (8.8)	166.7 (9.4)	<.001	
Weight (kg), mean (SD)		86.9 (14.6)	88.3 (13.6)	83.2 (18.0)	.25	
BMI (kg/m^2^), mean (SD)		29.2 (4.9)	28.6 (4.5)	30.2 (5.4)	.18	
**Coronary artery disease, n (%)**						
	One-vessel disease	33 (42)	14 (29)	19 (63)	.02	
	Two-vessel disease	26 (33)	18 (37)	8 (27)^b^	>.99	
	Three-vessel disease	20 (25)	17 (13)	3 (10)^b^	.36	
	ST-elevation myocardial infarction/non–ST-elevation myocardial infarction	63 (80)	36 (73)	27 (90)	.09	
**Treatment, n (%)**						
	Percutaneous coronary intervention performed	67 (85)	39 (80)	28 (93)	.12	
	Bypass performed	13 (16)	11 (22)	2 (7)	.12	
**Left ventricular ejection fraction, n (%)**						
	Normal (>50%)	56 (71)	35 (71)	21 (70)	>.99	
	Slightly reduced (41%-50%)	16 (20)	10 (21)^b^	6 (20)^b^	>.99	
	Moderately reduced (31%-40%)	7 (9)	4 (8)^b^	3 (10)^b^	>.99	
	Severely reduced (≤30%)	0 (0)	0 (0)^b^	0 (0)^b^	>.99	
Comorbidity index, median (range)^c^		1.9 (0-8)	1.6 (0-7)	2.3 (1-8)	.03	
**Education level, n (%)**						
	≤High school	69 (87)	45 (92)	24 (80)	>.99	
	>High school	10 (13)	4 (8)	6 (20)	>.99	
	Primary^d^	28 (37)	18 (39)	10 (33)	>.99	
	Secondary^e^	25 (33)	16 (35)	9 (30)	>.99	
	Abitur/Bachillerato^f^	13 (17)	8 (17)	5 (17)	>.99	
	University	10 (13)	4 (9)	6 (20)	>.99	
**Smoking status, n (%)**						
	Never	21 (27)	12 (24)	9 (30)	>.99	
	Former	34 (44)	27 (55)^b^	7 (24)	.001	
	Active	24 (30)	10 (20)	14 (47)	.57	

^a^*P* values were calculated using independent 2-sided *t* test (nonnormally distributed data were analyzed by Mann Whitney *U* test) and analysis of variance (nonnormally distributed variables were analyzed by Kruskal-Wallis rank sum test).

^b^*P*<.05 for within-group comparison.

^c^Comorbidity index was calculated according to the modified D’Hoore comorbidity index.

^d^Primary education is known as Hauptschule in Germany (DE) and educación primaria in Spain (ES).

^e^Secondary education is known as Realschule in Germany (DE) and educación secundaria obligatoria or vocational training in Spain (ES).

^f^Tertiary education is known as Abitur in Germany and Bachillerato in Spain.

### Digital Literacy and Current Usage of Technology

**Figure 1:**
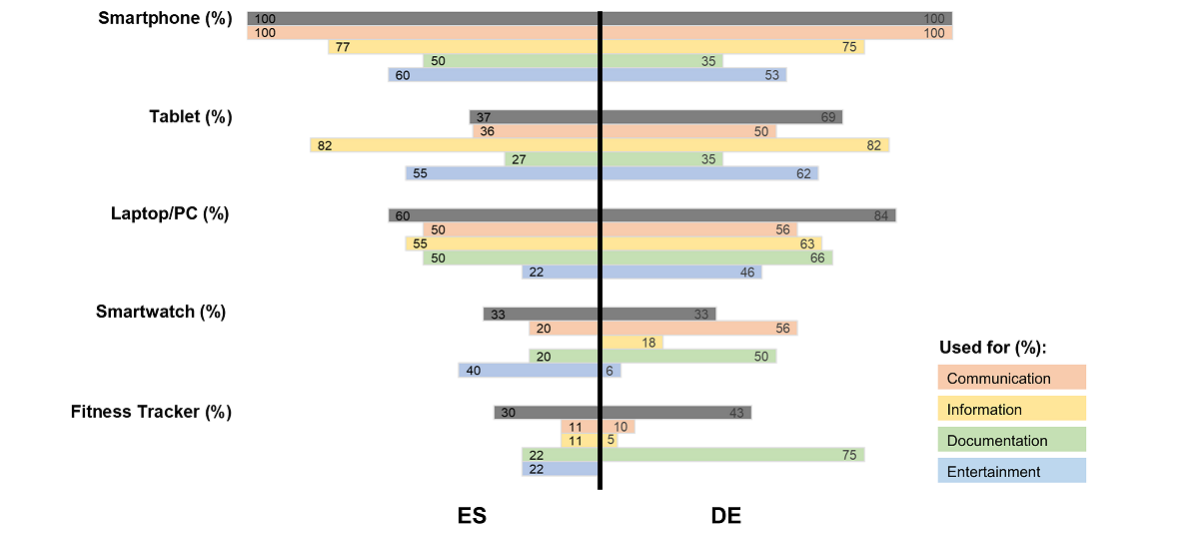
Current usage of technology on information and communication devices for different purposes, including health and fitness applications, by patients with coronary artery disease undergoing cardiac rehabilitation. DE: Germany; ES: Spain.

For the assessment of the use of technology among patients and their associated digital literacy, participants were asked what devices they owned, for which purpose the devices were used, and how experienced they were with health/fitness apps. All patients owned a smartphone, while a significantly lower proportion of Spanish patients owned a tablet (ES: 11/30, 37%; DE: 34/49, 69%; *P*=.005) (Figure 1). The majority of patients also owned a notebook or PC (ES: 18/30, 60%; DE: 25/30, 84%). Smartwatches (ES: 10/30, 33%; DE: 16/49, 33%) and fitness trackers (ES: 9/30, 30%; DE: 21/49, 43%) were used by a significant proportion of the participants with no differences between centers. Although smartphone, tablet, and notebook/PC were predominantly used for communication and information by the patients, a difference for smartwatch/fitness trackers was recorded in that up to 40% (12/30) of the Spanish patients used those devices also for entertainment. This was only reported by 6% (3/49) of the German patients (*P*=.06). Instead, 50% (25/49) of the German patients used wearables and associated apps for documentation (including physical activity), which was only reported by 20% (6/30) of the Spanish patients (*P*>.05). In terms of experience with automatic blood pressure monitors, 62% (49/79) of the patients reported their level of experience as “experienced” to “very experienced,” and 29% (23/79) and 13% (10/79) reported this level of experience for fitness trackers and health apps, respectively (Multimedia Appendix 1). Of note, more than 40% (32/79) of the patients reported at least some experience with health or fitness apps.

### Rating of CR Components

To assess how patients rated the importance of different CR components for disease management, we recorded their feedback on separate aspects of CR (using 5-point rating scales). Patients’ overall rating of the importance of CR components along the continuum of care for risk reduction was very high, including regular physical exercise (median 5, IQR 3-5), healthy diet (median 5, IQR 3-5), stress management (median 5, IQR 1-5), smoking cessation (median 5, IQR 1-5), optimal medication (median 5, IQR 3-5), motivation for lifestyle changes (median 5, IQR 3-5), and overall risk factor management (median 5, IQR 2-5), with no significant difference between the 2 centers. Patients also rated their individual need for support during phase III CR maintenance in the beforementioned areas, revealing large interindividual differences with all items ranging from 1 to 5. In general, patients expressed a high need for support for regular physical exercise (median 4, range 1-5), less need for support for smoking cessation (median 1, range 1-5; only active smokers were asked), and less support for healthy diet (median 3, range 1-5), stress management (median 3, range 1-5), medication (median 3, range 1-5), motivation for lifestyle changes (median 3, range 1-5), and risk factor management (median 3, range 1-5). Of note, for each item except from smoking cessation, 35%-50% of the patients indicated a high need for support (≥4) to achieve their long-term health goals, suggesting a need for individualized care. The subgroup of patients expressing low perceived smoking cessation support needs was analyzed further to investigate if it includes patients with high-risk phenotypes. However, this analysis did not suggest an elevated risk for these patients, as age, sex, BMI, disease severity (bypass performed [yes/no]), and comorbidity index were similar to those of the group of smokers indicating need for smoking cessation support.

### Rating of eHealth Components to Assist in Phase III CR Maintenance

**Figure 2:**
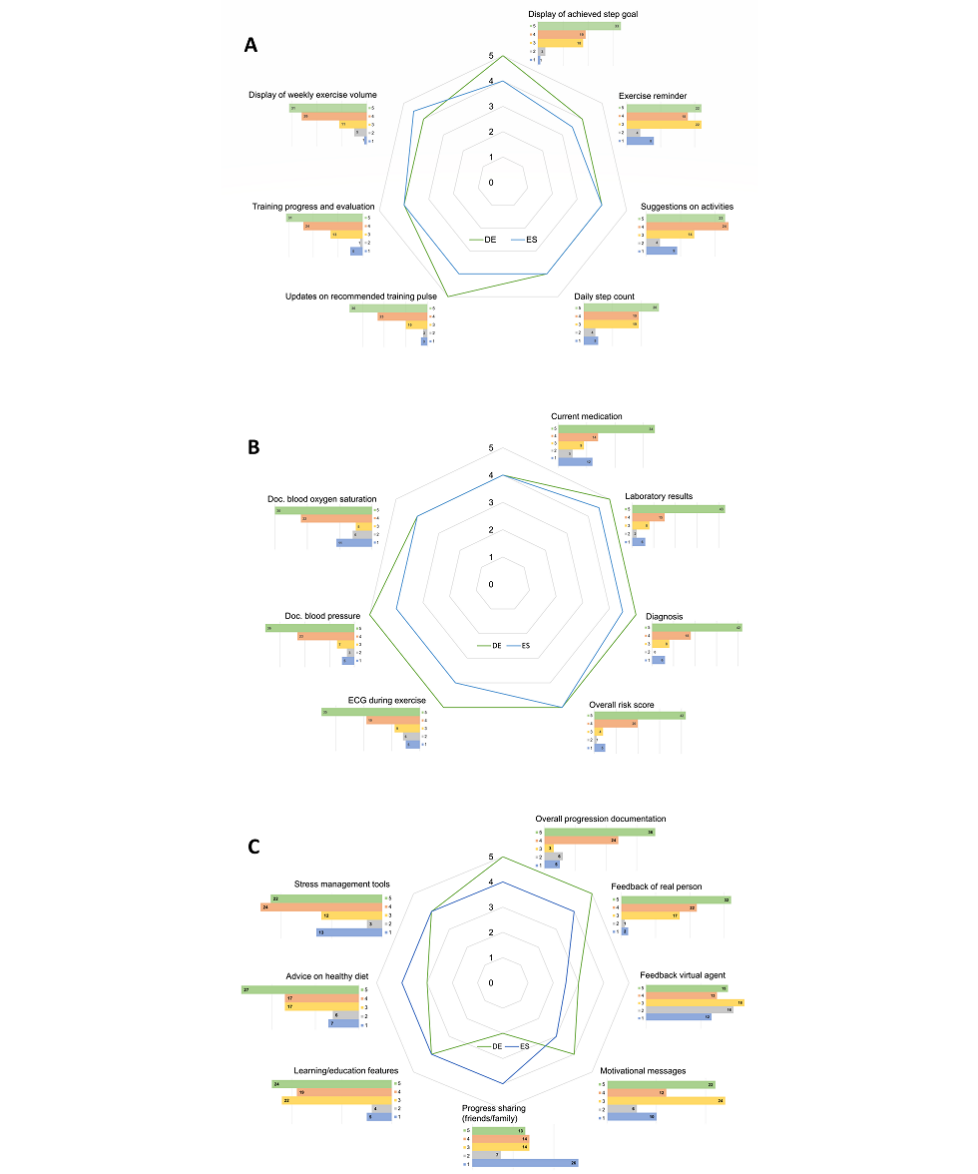
Results of the patient survey: rating of cardiac rehabilitation components. Patient-reported perceived importance of different cardiac rehabilitation components to assist during phase III cardiac rehabilitation maintenance. (A) Components to support regular physical activity, (B) components informing on diagnosis, clinical/laboratory parameters, and risk score, (C) other cardiac rehabilitation–related topics/functions. 5-point Likert scale (1=unimportant/not useful; 5=very important/very useful). DE: Germany; Doc.: documentation; ECG: electrocardiogram; ES: Spain.

Overall, 71% (56/79) of the patients reported that they considered technology, including mobile apps, to be helpful in maintaining health goals after phase II CR. To investigate the specific needs and expectations for an eHealth system to assist in phase III CR maintenance, we asked patients about the features that would be the most helpful for reaching their individual health goals if they were free to choose from a predefined set of options. The presented features were selected by the TIMELY investigators involving cardiologists, rehabilitation experts, behavioral change experts, sports scientists, and by considering recent literature on eHealth in CR [6]. Selected features were grouped into 3 categories for the presentation of results, including exercise-related features, clinical/medical components, and motivational/other features (Figure 2) and were analyzed for differences between nationality, age groups, and men versus women. No significant differences between nationalities were detected for exercise-related features or medical-related entities. In the domain of other CR components, overall progression documentation was significantly rated as more useful/more needed by German patients (median 5, range 1-5) than by Spanish patients (median 4, range 1-5; *P*<.001). German patients also rated “individual feedback of a real person” more useful than Spanish patients (median 5, range 1-5 vs median 4, range 3-5; *P*=.005, respectively). With respect to motivational features, Spanish patients rated the possibility to “share progress with friends and family” as more useful than German patients (median 4, range 1-5 vs median 2, range 1-5]; *P*=.02, respectively). When asked about the preferred frequency for motivational messages, only 5% of the patients answered “several times a day.” Approximately 27% (21/79) preferred to receive messages once a day, 26% (20/79) every other day, and 9% (7/79) did not want to receive messages. Approximately 32% (25/79) indicated that they would prefer a flexible schedule for messages. Of note, no differences in preference for any suggested features were detected between women and men or among age groups. However, the score for most items ranged from 1 to 5, highlighting that perceived usefulness of potential eHealth features differs substantially between individuals.

### Factors Associated With Acceptance of eHealth in CR Maintenance

To investigate the factors associated with the acceptance of eHealth, we used questionnaires to analyze factors such as sex, age, clinical data, educational as well as psychological factors. Questionnaires involved LOT-R for optimism/pessimism, AAQ-2 for psychological flexibility, DS-14 for social inhibition, and PANAS for positive/negative affectivity. Education level was not associated with the acceptance of eHealth components (Table 2). No differences were observed with regard to acceptance between women and men, but younger age was significantly associated with more acceptance of monitoring devices (*P*=.03), while only a tendency was seen for willingness to use a mobile app (*P*=.11). Of note, only 6% (3/49) of the patients who accepted eHealth indicated they would likely not use eHealth components because of privacy concerns, and 8% (4/49) of the patients did not like the idea of being monitored. Although multivariate linear regression analysis did not identify a combination of factors associated with eHealth acceptance, naïve Bayes classification suggested that eHealth acceptance may potentially be predicted based on younger age, a lower AAQ-2 score indicating psychological flexibility, and the index event (having experienced myocardial infarction). Willingness to use a mobile app was predicted with an overall accuracy of 97.9% (using age and AAQ-2), and the acceptance of monitoring devices was predicted with an overall accuracy of 91.7% (using age, AAQ-2, and myocardial infarction). However, validation in an independent data set was not performed.

**Table 2:** Predictors of eHealth acceptance among patients with coronary artery disease^a^.

			Would use mobile app for support								Would use devices for monitoring						
			Yes/maybe (n=42)		No (n=7)		*P* value			Yes/maybe (n=40)			No (n=9)			*P* value	
**Sex, n (%)**								.62									>.99
	Female	8 (80)		2 (20)					8 (80)			2 (20)					
	Male	34 (87)		5 (13)					32 (82)			7 (18)					
**Age (years), n (%)**								.11									.03
	≥57 years	21 (78)		6 (22)					19 (70)			8 (30)					
	<57 years	21 (95)		1 (5)					19 (70)			1 (5)					
**Education^b^, n (%)**								>.99									.66
	≥High school	11 (92)		1 (8)					11 (92)			1 (8)					
	<High school	29 (85)		5 (15)					28 (76)			6 (24)					
**LOT-R^c^, median (IQR)**																	
	Optimism	4 (0-9)		4 (2-6)		.79			4 (0-9)			4 (2-6)			.61		
	Pessimism	7 (2-12)		4 (3-7)		.11			6 (2-12)			7 (3-9)			.76		
AAQ-2^d^ flexibility, median (IQR)			14 (7-36)		19 (7-27)		.92			10 (7-36)			15 (7-27)			.23	
**DS-14^e^, median (IQR)**																	
	Negative affectivity	10 (1-28)		13 (4-19)		.69			11 (1-28)			10 (2-21)			.80		
	Social inhibition	12 (0-20)		11 (7-16)		.81			12 (0-20)			8 (7-18)			.23		
**PANAS^f^, median (IQR)**																	
	Positive affect	3 (1-4)		3 (2-5)		.69			3 (1-4)			3 (2-5)			.77		
	Negative affect	2 (1-4)		2 (1-4)		.47			2 (1-4)			2 (1-4)			.47		

^a^Data are given as n (%) and median and range. Patients were asked if they would use a mobile app for their cardiac rehabilitation maintenance support and if they would use monitoring devices (eg, blood pressure monitor, electrocardiogram, activity tracker) during maintenance. Options provided were yes/maybe or no. Between-group comparison was performed using chi-square test or Mann-Whitney *U* test.

^b^Three missing. Only German patients (n=40) were involved.

^c^LOT-R: Revised Life Orientation Test; 2 dimensions; range 0-12 (higher = larger optimism/pessimism).

^d^AAQ-2: Acceptance and Action Questionnaire version 2; range 7-49 (higher = greater psychological inflexibility).

^e^DS-14: Type D scale for social inhibition; 2 dimensions; range 0-28 (higher = larger negative affectivity/social inhibition).

^f^PANAS: Positive and Negative Affect Schedule; 2 dimensions; range 0-10 (higher = larger affect).

## Discussion

### Principal Findings

This study aimed to define patients’ needs and expectations for eHealth-based CR to assist them during the lifelong maintenance phase. A Living Lab approach was used for German and Spanish patients with CAD to characterize their use of technology, their preferences and rating of importance for different components of a future eHealth solution for CR maintenance, as well as their general willingness to use eHealth. In brief, our main findings are (1) patients with CAD appreciated being involved in the planning of a future eHealth system, and they had sufficient levels of digital literacy, (2) patients rated the importance of CR components along the continuum of care for risk reduction as very high, (3) 71% (56/79) of the patients expected that technology could help them to maintain health goals after center-based CR, and (4) a large intraindividual heterogeneity was detected in terms of reported needs and perceived usefulness for different eHealth components.

CAD is a chronic disease, necessitating innovative approaches for effective management and support over the lifelong maintenance phase after successful intervention and rehabilitation [1-3]. In recent years, telemedicine and eHealth solutions have emerged as promising tools for improving the care of patients with CAD [6]. In this regard, eHealth has already been shown to be an effective alternative to phase II CR, and a recent meta-analysis suggested that telehealth-based phase II CR may be even superior to center-based programs at least for enhancing physical activity levels [28-30]. In addition, eHealth may have the potential to involve a large number of patients since it may also be an option for patients who cannot or do not want to attend a center-based CR. In terms of cost efficiency, Frederix et al [30] estimated that a 6-month internet-based program consisting of exercise training with telemonitoring support, text messages, and web service can be cost-efficient for up to 2 years after the end of the intervention [30]. However, the development of eHealth solutions tailored for patients with CAD requires a dynamic and patient-centered approach since low user acceptance is one of the largest barriers for success of these solutions. The European Society of Cardiology e-Cardiology Working Group reported that digital health developments are often technically driven and not based on the needs and expectations of patients, thereby calling for cocreation with patient involvement in the design [15]. The European Society of Cardiology position paper strongly emphasized that patient-related barriers and user characteristics may hinder the large-scale deployment of eHealth services. Thus, the TIMELY project includes a Living Lab as means to involve patients and patient organizations, and our analyses reflect part of this patient-centered approach.

Per definition, Living Labs represent open innovation ecosystems to cocreate, assess, and refine innovative (technical) solutions [19]. To achieve a user-centric design, Living Labs prioritize the engagement of patients together with health care professionals to ensure that the resulting applications align with the needs, preferences, and challenges faced by the specific needs of a patient group. It is however important to place Living Labs in authentic settings, as implemented in this study, where patients with CAD undergoing center-based phase II CR are involved. These patients had received comprehensive information on the etiology and treatment of their disease as well as lifestyle factors that modify CAD. The majority of the involved patients indicated that they liked the approach and appreciated being involved in the conception and development of an eHealth solution to assist them during the maintenance phase even though some indicated that too much effort might keep them from using such a solution. In terms of predictors of eHealth use, previous research on sociodemographic factors among US adult internet users suggested that patients with lower education levels had lower odds of using certain features, including web-based tracking of personal health information, using a website to support physical activity, or downloading health information to a mobile device [31]. That study also indicated that being female was a predictor of eHealth use across health care and user-generated content, while age influenced health information–seeking [31]. In comparison, our data also suggest that younger age was associated with the indicated acceptance of technology, but women were as likely as men to accept eHealth for managing their disease, and the education level was not identified as a predictor. These findings might be based on the fact that smartphones, device hardware, and mobile apps are rapidly advancing, and daily exposure lowers the barriers for patients to use technology [32]. Although our study was performed among a selected group of patients with CAD participating in a prospective study, it is interesting to compare our cohort also in terms of the necessary hardware availability, that is, smartphone ownership in this patient group in general. Between 2019 and 2020, a large cross-sectional study among cardiac inpatients in Australia reported a high frequency of smartphone ownership (85%-89%) among patients aged 50-69 years and lower ownership (~60%) in patients aged 70-79 years [33]. In our sample (mean age 57 years, SD 9 years), every patient owned a smartphone and one-third also used activity trackers/smartwatches, which might also be explained by the differences between countries (Australia vs Germany/Spain). Percentage of technology ownership as well as usage and expectations for eHealth were not different between Germany and Spain, even though the Spanish population was significantly older (*P*=.001) and clinical characteristics differed to some extent. Further, CR in Spain is based on outpatient care, which, while equally effective in terms of reaching the main CR outcomes, could have affected the estimated need for eHealth in this population. Of the analyzed psychological factors, only psychological flexibility showed some predictive value for eHealth acceptance. This result partly contradicts previous findings among older (>60 years) residents of Hong Kong, wherein optimism was significantly related to perceived eHealth usefulness [34]. To what extent these differences are caused by differences in age or cultural background warrant further investigations.

State-of-the-art digital health care programs face numerous technical and interoperability hurdles that make implementation difficult. This includes transmitting physiological measurements from ECGs and blood pressure monitors as well as data from activity trackers and other wearables to a centralized platform. Respective solutions rely on wireless networks; different hardware, software, and algorithms for capturing and processing data; as well as connected dashboards. Challenges include system reliability, data quality, interoperability, and overall, the highest level of data security. We have not asked the involved patients about their opinions on system availability and stability, as these aspects as well as data security and privacy need to meet the highest standards as conditio sine qua non when providing eHealth to patients. However, information regarding these aspects needs to be provided to patients in sufficient detail, since privacy-related concerns represent considerable barriers [15,35]. These technical requirements and interdependencies result in high costs for any eHealth solution targeting to improve patients’ self-care. Foreseen functionalities should thus not only be based on current guidelines but should be aligned with patient needs and expectations. This study shows that patients with CAD expected considerable merit in the documentation and availability of their diagnosis, laboratory results, and current medication—all details that would be part of an electronic health record. Patients also showed interest in their overall risk score, which TIMELY will base on a biomarker score to predict the 10-year mortality risk [36,37]. The majority of patients rated the usefulness of blood pressure and ECG monitors as high or very high. Functionalities related to support daily physical activities and physical exercise were perceived as (very) useful, with most patients indicating a high need for progress documentation and regular updates on personalized training recommendations. This observation is relevant since commercial activity trackers have been reported to significantly increase the daily step count and aerobic capacity in patients undergoing CR [38,39], and a considerable number of patients were already relying on commercial solutions, which, however, do not always provide the necessary level of data protection and have not been tested sufficiently in patient populations. Functionalities related to other important parts of CR, including smoking cessation, stress management, advice on heart-healthy eating, as well as self-education, were perceived as less useful or rated neutral, likely depending on the individual perceived needs of the patients. This aspect was pronounced for smoking cessation, which was perceived as an important part of CR, but 50% of the smokers indicated that they did not want support with this health-related aspect.

### Limitations

Although reporting on 2 samples of participants undergoing CR from Germany and Spain with cultural and socioeconomic differences is a strength of this study, this report may be affected by the potential study selection bias since patients participating in scientific research studies differ in terms of motivational aspects. However, our sample population did not differ with respect to the sociodemographic characteristics of the samples of patients with CAD undergoing CR who were analyzed in previous reports [22]. It should be noted that health literacy, a central factor in eHealth usage and a pivotal determinant of health in general, is a complex construct and was not assessed in all dimensions in our study population. The results of naïve Bayes classification should be interpreted with care since validation in an independent data set was not performed. The timepoint and situation of this survey may also have affected the results since patients may answer differently when asked in their home environment or with greater time interval after an acute event. Focus groups may allow for more and detailed information on the reasoning underlying the reported answers to this guided survey, and the results of focus groups within TIMELY will be reported elsewhere.

### Conclusion

This survey involving patients undergoing CR in Germany and Spain revealed that eHealth for CR maintenance should emphasize on support for regular physical activity and physical exercise, including patient feedback on achievements and renewal of training recommendations. Devices for physiological measurements, including blood pressure and ECG monitors, were considered useful, and most patients expressed a need for the documentation of diagnosis, medication, and laboratory results in terms of an electronic health record. In general, the patients who took part in this project showed a sufficient level of digital literacy and current usage of technology to make good use of even more advanced eHealth solutions. Although only minor differences were observed among Spanish and German patients as well as between female and male patients and educational status did not appear to be a contributing factor, it is crucial to note substantial variability in patients’ individual needs and expectations. Consequently, eHealth solutions should prioritize personalization to enhance user acceptance. Next steps of the TIMELY Living Lab will involve analyses of details on the implementation of the individual CR functionalities and feedback on the mobile app design.

## Appendix

Multimedia Appendix 1 Details of the survey.

## Abbreviations

AAQ-2: Acceptance and Action Questionnaire version 2
CAD: coronary artery disease
CR: cardiac rehabilitation
DE: country code for Germany
DS-14: Type D scale for social inhibition
ECG: electrocardiogram
ES: country code for Spain
LOT-R: Revised Life Orientation Test
PANAS: Positive and Negative Affect Schedule

## Data Availability

The data generated during this study are available from the corresponding author upon reasonable request.
