# Supplementary material for: Living Lab Data of Patient Needs and Expectations for eHealth-Based Cardiac Rehabilitation in Germany and Spain From the TIMELY Study: Cross-Sectional Analysis
Source: J Med Internet Res. 2024 Feb 22;26:e53991. doi: 10.2196/53991 (PMC10921324; doi:10.2196/53991)
Supplement: Multimedia Appendix 1 [file jmir_v26i1e53991_app1.docx]

**Multimedia Appendix 1**

| **Part 1: Rehabilitation components and their importance** |  |  |  |  |
| --- | --- | --- | --- | --- |
| **Q1:**  *"Have you ever participated in medical rehabilitation before your currant stay?"* | Overall [N=79] | DE [N=49] | ES [N=30] |  |
|  |  |  |  |  |
|  |  |  |  |  |
| *Yes, outpatient for less than 6 weeks* | 16 [20.3] | 3 [3.8] | 13 [16.5] |  |
| *Yes, outpatient for more than 6 weeks* | 11[13.9] | 2 [2.5] | 9 [11.4] |  |
| *Yes, inpatient for up to 4 weeks* | 16 [20.3] | 16 [20.3] | 0 [0] |  |
| *No* | 32 [40.5] | 24[30.4] | 8 [10.1] |  |
| *Other* | 4 [5.1] | 4 [5.1] | 0 [0] |  |
| **Q2:** *"What was the reason for the medical rehabilitation?" §* * |  |  |  |  |
|  |  |  |  |  |
|  |  |  |  |  |
| *Cardiac* | 23 [50] | 13 [54.2] | 10 [45.5] |  |
| *Orthopaedic* | 26 [56.2] | 8 [33.3] | 18 [81.8] |  |
| *Other* | 4 [8] | 3 [12.5] | 1 [4.5] |  |
| **Q3a:** *"How important do you think the following areas are for reducing your risk/ cardiac patients' risk after rehabilitation?"*  ***Regular exercise*** |  |  |  |  |
|  |  |  |  |  |
|  |  |  |  |  |
| *unimportant* | 0 [0] | 0 [0] | 0 [0] |  |
| *less important* | 0 [0] | 0 [0] | 0 [0] |  |
| *neutral* | 2 [2.5] | 0 [0] | 2 [6.7] |  |
| *important* | 13 [16.5] | 6 [12.2] | 7 [23.3] |  |
| *very important* | 64 [81] | 43 [87.7] | 21 [70] |  |
| **Q3b: *Healthy diet*** |  |  |  |  |
|  |  |  |  |  |
|  |  |  |  |  |
| *unimportant* | 0 [0] | 0 [0] | 0 [0] |  |
| *less important* | 0 [0] | 0 [0] | 0 [0] |  |
| *neutral* | 5 [6.3] | 4 [8.2] | 1 [3.3] |  |
| *important* | 18 [22.8] | 10 [20.4] | 8 [26.7] |  |
| *very important* | 56 [70.9] | 35 [71.4] | 21 [70] |  |
| **Q3c: *Stress management*** |  |  |  |  |
|  |  |  |  |  |
|  |  |  |  |  |
| *unimportant* | 1 [1.3] | 1 [2] | 0 [0] |  |
| *less important* | 0 [0] | 0 [0] | 0 [0] |  |
| *neutral* | 7 [8.9] | 4 [8.2] | 3 [10] |  |
| *important* | 26[32.9] | 18 [36.7] | 8 [26.7] |  |
| *very important* | 45 [57] | 26 [53.1] | 19 [63.3] |  |
| **Q3d: *Smoking cessation ^#^*** |  |  |  |  |
|  |  |  |  |  |
|  |  |  |  |  |
| *unimportant* | 7 [10.4] | 3 [8.1] | 4 [13.3] |  |
| *less important* | 0 [0] | 0 [0] | 0 [0] |  |
| *neutral* | 1 [1.5] | 1 [2.7] | 0 [0] |  |
| *important* | 8 [11.9] | 6 [16.2] | 2 [6.7] |  |
| *very important* | 51 [76.1] | 27 [73] | 24 [80] |  |
| **Q3e: *Medication*** |  |  |  |  |
|  |  |  |  |  |
|  |  |  |  |  |
| *unimportant* | 0 [0] | 0 [0] | 0 [0] |  |
| *less important* | 0 [0] | 0 [0] | 0 [0] |  |
| *neutral* | 6 [7.6] | 4 [8.2] | 2 [6.7] |  |
| *important* | 14 [17.7] | 11 [22.4] | 3 [10] |  |
| *very important* | 59 [74.7] | 34 [69.4] | 25 [83.3] |  |
| **Q3f: *Motivation for lifestyle changes*** |  |  |  |  |
|  |  |  |  |  |
|  |  |  |  |  |
| *unimportant* | 0 [0] | 0 [0] | 0 [0] |  |
| *less important* | 0 [0] | 0 [0] | 0 [0] |  |
| *neutral* | 5 [6.3] | 5 [10.2] | 0 |  |
| *important* | 27 [34.2] | 12 [24.5] | 15 [50] |  |
| *very important* | 47 [59.5] | 32 [65.3] | 15 [50] |  |
| **Q3g: *Overall risk factor management*** |  |  |  |  |
|  |  |  |  |  |
|  |  |  |  |  |
| *unimportant* | 0 [0] | 0 [0] | 0 [0] |  |
| *less important* | 1 [1.3] | 1 [2] | 0 [0] |  |
| *neutral* | 6 [7.6] | 2 [4.1] | 4 [13.3] |  |
| *important* | 29 [36.7] | 19 [38.8] | 10 [33.3] |  |
| *very important* | 43 [54.4] | 27 [55.1] | 16 [53.3] |  |
| **Q4a:** *"In which area(s) would you like to receive additional support after rehabilitation? (To maintain health goals)"* ***Regular exercise*** |  |  |  |  |
|  |  |  |  |  |
|  |  |  |  |  |
| *No support* | 17 [21.5] | 12 [24.5] | 5 [16.7] |  |
| *less support* | 6 [7.6] | 4 [8.2] | 2 [6.7] |  |
| *neutral/ unsure* | 13 [16.5] | 8 [16.3] | 5 [16.7] |  |
| *some support* | 15 [19] | 9 [18.4] | 6 [20] |  |
| *Strong support* | 28 [35.4] | 16 [32.6] | 12 [40] |  |
| **Q4b: *Healthy diet*** |  |  |  |  |
|  |  |  |  |  |
|  |  |  |  |  |
| *No support* | 20 [25.3] | 17 [34.7] | 3 [10] |  |
| *less support* | 6 [7.6] | 4 [8.2] | 2 [6.7] |  |
| *neutral/ unsure* | 22 [22.8] | 13 [26.5] | 5 [16.7] |  |
| *some support* | 15 [19] | 5 [10.2] | 10 [33.3] |  |
| *Strong support* | 20 [25.3] | 10 [20.4] | 10 [33.3] |  |
| **Q4c: *Stress management*** |  |  |  |  |
|  |  |  |  |  |
|  |  |  |  |  |
| *No support* | 15 [19] | 12 [24.5] | 3 [10] |  |
| *less support* | 7 [8.9] | 4 [8.2] | 3[10] |  |
| *neutral/ unsure* | 18 [22.8] | 12 [24.5] | 6 [20] |  |
| *some support* | 23 [29.1] | 13 [26.5] | 10 [33.3] |  |
| *Strong support* | 16 [20.3] | 8 [16.3] | 8 [26.7] |  |
| **Q4d: *Smoking cessation ^#^*** |  |  |  |  |
|  |  |  |  |  |
|  |  |  |  |  |
| *No support* | 31 [52.5] | 16 [55.2] | 15 [50] |  |
| *less support* | 1 [1.7] | 1 [3.5] | 0 [0] |  |
| *neutral/ unsure* | 7 [11.9] | 1 [3.5] | 6 [20] |  |
| *some support* | 5 [8.5] | 3 [10.5] | 2 [6.7] |  |
| *Strong support* | 15 [25.4] | 8 [27.6] | 7[23.3] |  |
| **Q4e: *Medication*** |  |  |  |  |
|  |  |  |  |  |
|  |  |  |  |  |
| *No support* | 34 [43] | 23 [46.9] | 11 [36.6] |  |
| *less support* | 5 [6.3] | 2 [4.1] | 3 [10] |  |
| *neutral/ unsure* | 11 [13.9] | 9 [18.4] | 2 [6.7] |  |
| *some support* | 13[16.5] | 7 [14.3] | 6 [20] |  |
| *Strong support* | 16[20.3] | 8 [16.3] | 8 [26.7] |  |
| **Q4f: *Motivation for lifestyle changes*** |  |  |  |  |
|  |  |  |  |  |
|  |  |  |  |  |
| *No support* | 19 [24.1] | 14 [28.6] | 5 [16.7] |  |
| *less support* | 7 [8.9] | 4 [8.2] | 3 [10] |  |
| *neutral/ unsure* | 21 [26.6] | 16 [32.6] | 5 [16.7] |  |
| *some support* | 18 [22.8] | 10 [20.4] | 8 [26.7] |  |
| *Strong support* | 14 [17.7] | 5 [10.2] | 9 [30] |  |
| **Q4g: *Overall risk factor management*** |  |  |  |  |
|  |  |  |  |  |
|  |  |  |  |  |
| *No support* | 14 [17.7] | 11 [22.5] | 3 [10] |  |
| *less support* | 4 [5.1] | 2 [4.1] | 2 [6.7] |  |
| *neutral/ unsure* | 22 [27.8] | 12 [24.5] | 10 [33.3] |  |
| *some support* | 23 [29.1] | 17 [34.7] | 6 [20] |  |
| *Strong support* | 16 [20.3] | 7 [14.3] | 9 [30] |  |
| **Q5:** *"Have any technical aids, such as a fitness bracelet/watch been recommended to you during rehabilitation?"* |  |  |  |  |
|  |  |  |  |  |
|  |  |  |  |  |
| *Yes* | 40 | 25 [51] | 15 [50] |  |
| *No* | 36 | 21 [42.9] | 15 [50] |  |
| *I do not remember* | 3 | 3 [6.1] | 0 [0] |  |
| **Q6:** *"Would you say that you have/ had to deal with new technical aspects/ solutions in your job?"* |  |  |  |  |
|  |  |  |  |  |
|  |  |  |  |  |
| *Yes* | 37 [47.4] | 23 [46.9] | 14 [46.7] |  |
| *No* | 25 [32.1] | 11 [22.5] | 14 [46.7] |  |
| *I do not remember* | 16 [20.5] | 14 [28.6] | 2 [6.7] |  |
| **Part 2: Digital literacy/ use of digital technology** |  |  |  |  |
| **Q7:**  *"Are you skilled with technology, do you have a technical know-how?"* |  |  |  |  |
|  |  |  |  |  |
|  |  |  |  |  |
| *No* | 15 [19] | 9 [18.4] | 6 [20] |  |
| *Yes* | 30 [38] | 23 [47] | 7 [23.3] |  |
| *Depends on the device* | 34 [43] | 17 [34.6] | 17 [56.7] |  |
| **Q8:** *"Are you open minded towards new technology?"* ^*^ |  |  |  |  |
|  |  |  |  |  |
|  |  |  |  |  |
| *No* | 12 [15.2] | 6 [12.3] | 6 [20] |  |
| *Yes* | 58 [73.4] | 35 [71.4] | 23 [76.7] |  |
| *Depends on the device* | 9 [11.4] | 8 [16.3] | 1 [3.3] |  |
| **Q9a:** *"Do you own a...?"*  ***Smartphone*** |  |  |  |  |
|  |  |  |  |  |
|  |  |  |  |  |
| *No* | 0 [0] | 0 [0] | 0 [0] |  |
| *Yes* | 79 [100] | 49 [100] | 30 [100] |  |
| **Q9b: *Tablet*** |  |  |  |  |
|  |  |  |  |  |
|  |  |  |  |  |
| *No* | 34 [43] | 15 [30.6] | 19 [63.3] |  |
| *Yes* | 45 [57] | 34 [69.4] | 11 [36.7] |  |
| **Q9c: *Laptop/ Pc*** |  |  |  |  |
|  |  |  |  |  |
|  |  |  |  |  |
| *No* | 20 [25.3] | 8 [16.3] | 12 [40] |  |
| *Yes* | 59 [74.7] | 41 [83.7] | 18 [60] |  |
| **Q9d: *Smartwatch*** |  |  |  |  |
|  |  |  |  |  |
|  |  |  |  |  |
| *No* | 53 [67.1] | 33 [67.4] | 20 [67.7] |  |
| *Yes* | 26 [32.9] | 16 [32.6] | 10 [33.3] |  |
| **Q9e: *Fitness Tracker*** |  |  |  |  |
|  |  |  |  |  |
|  |  |  |  |  |
| *No* | 49 [62] | 28 [57.1] | 21 [70] |  |
| *Yes* | 30 [38] | 21 [42.9] | 9 [30] |  |
| **Q10a:** "Do you have experience using a …? ***Automatic blood pressure monitor*** |  |  |  |  |
|  |  |  |  |  |
|  |  |  |  |  |
| *No experience* | 18 [22.8] | 12 [24.5] | 6 [20] |  |
| *Limited experience* | 3 [3.8] | 1 [2] | 2 [6.7] |  |
| *Some experience* | 9 [11.4] | 5 [10.2] | 4 [13.3] |  |
| *Experienced* | 11 [13.9] | 3 [6.1] | 8 [26.7] |  |
| *Very experienced* | 38 [48.1] | 28 [57.2] | 10 [33.3] |  |
| **Q10b: *Fitness tracker*** |  |  |  |  |
|  |  |  |  |  |
|  |  |  |  |  |
| *No experience* | 43 [94.1] | 29 [59.2] | 14 [46.7] |  |
| *Limited experience* | 4 [5.1] | 1 [2] | 3 [10] |  |
| *Some experience* | 9 [11.4] | 5 [10.2] | 4 [13.3] |  |
| *Experienced* | 8 [10.1] | 2 [4.1] | 6 [20] |  |
| *Very experienced* | 15 [19] | 12 [24.5] | 3 [10] |  |
| **Q10c:** ***Glucose monitor/ insulin pump*** |  |  |  |  |
|  |  |  |  |  |
|  |  |  |  |  |
| *No experience* | 75 [94.9] | 47 [95.2] | 28 [93.3] |  |
| *Limited experience* | 1 [1.3] | 0 [0] | 1 [3.3] |  |
| *Some experience* | 0 [0] | 0 [0] | 0 [0] |  |
| *Experienced* | 1 [1.3] | 0 [0] | 1 [3.3] |  |
| *Very experienced* | 2 [2.5] | 2 [4.1] | 0 [0] |  |
| **Q10d: *Health/ fitness apps*** |  |  |  |  |
|  |  |  |  |  |
|  |  |  |  |  |
| *No experience* | 47 [59.5] | 30 [61.22] | 17 [56.7] |  |
| *Limited experience* | 6 [7.6] | 5 [10.2] | 1 [3.3] |  |
| *Some experience* | 16 [20.3] | 9 [18.4] | 7 [23.3] |  |
| *Experienced* | 9 [11.4] | 4 [8.2] | 5 [16.7] |  |
| *Very experienced* | 1 [1.3] | 1 [2] | 0 [0] |  |
| **Part 3: Acceptance of eHealth and preferences** |  |  |  |  |
| **Q11:**  *"In general, do you believe that technology could help you maintain your health goals after rehabilitation?"* |  |  |  |  |
|  |  |  |  |  |
|  |  |  |  |  |
| *No* | 10 [12.7] | 5 [10.2] | 5 [16.7] |  |
| *Yes* | 54[68.4] | 37 [75.5] | 17 [56.7] |  |
| *Unsure, do not know how* | 15 [19] | 7 [14.3] | 8 [26.7] |  |
| **Q12:** *"Would you use a mobile app to document your rehabilitation progress and possibly stay in contact with your rehabilitation team?"* |  |  |  |  |
|  |  |  |  |  |
|  |  |  |  |  |
| *No* | 13 [16.7] | 7 [14.3] | 6 [20] |  |
| *Yes* | 54[69.2] | 34 [69.4] | 20 [66.7] |  |
| *I do not know* | 12 [15.2] | 8 [16.3] | 4 [13.3] |  |
| **Q13:** *"Would you accept an offer of a free technical solution consisting of a blood pressure monitor, fitness tracker and ECG patch connected to a medical care system that would allow you and the rehabilitation team to monitor your progress and risk factors?"* |  |  |  |  |
|  |  |  |  |  |
|  |  |  |  |  |
| *No* | 10 [12.7] | 9 [18.4] | 1 [3.3] |  |
| *Yes* | 61 [77.2] | 39 [79.6] | 22 [73.3] |  |
| *Maybe* | 8 [10.1] | 1 [2] | 7 [23.3] |  |
| **Q14a:** *"Please rate the usefulness in regard to reaching your health goals of the following features…"* ***Display of archived steps to goal*** |  |  |  |  |
|  |  |  |  |  |
|  |  |  |  |  |
| *Not useful* | 1 [1.4] | 1 [2.3] | 0 [0] |  |
| *Less useful* | 2 [2.7] | 2 [4.5] | 0 [0] |  |
| *neutral* | 18 [24.3] | 7 [15.9] | 11 [36.7] |  |
| *Useful* | 19 [25.7] | 8 [18.2] | 11 [36.7] |  |
| *Very useful* | 34 [45.9] | 26 [59.1] | 8 [26.7] |  |
| **Q14b: *Motivational messages*** |  |  |  |  |
|  |  |  |  |  |
|  |  |  |  |  |
| *Not useful* | 10 [13.5] | 6 [13.6] | 4 [13.3] |  |
| *Less useful* | 6 [8.1] | 2 [4.5] | 4 [13.3] |  |
| *neutral* | 23 [31.1] | 11 [25] | 12 [40] |  |
| *Useful* | 12 [16.2] | 7 [15.9] | 5 [16.7] |  |
| *Very useful* | 23 [31.1] | 18 [40.9] | 5 [16.7] |  |
| **Q14c: *Exercise reminders*** |  |  |  |  |
|  |  |  |  |  |
|  |  |  |  |  |
| *Not useful* | 8 [10.8] | 6 [13.6] | 2 [6.7] |  |
| *Less useful* | 3 [4.1] | 3 [6.8] | 0 [0] |  |
| *neutral* | 22 [2.97] | 9 [20.4] | 13 [43.3] |  |
| *Useful* | 18 [24.3] | 12 [27.3] | 6 [20] |  |
| *Very useful* | 24 [31.9] | 14 [31.8] | 9 [30] |  |
| **Q14d: *Suggestions about activities*** |  |  |  |  |
|  |  |  |  |  |
|  |  |  |  |  |
| *Not useful* | 9 [12.2] | 7 [15.9] | 2 [6.7] |  |
| *Less useful* | 3 [4.1] | 2 [4.5] | 1 [3.3] |  |
| *neutral* | 14 [18.9] | 6 [13.6] | 8 [26.7] |  |
| *Useful* | 24 [32.4] | 14 [31.8] | 10 [33.3] |  |
| *Very useful* | 24 [32.4] | 15 [34.1] | 9 [30] |  |
| **Q14e: *Overall progress documentation*** |  |  |  |  |
|  |  |  |  |  |
|  |  |  |  |  |
| *Not useful* | 4 [5.4] | 1 [2.3] | 3 [10] |  |
| *Less useful* | 6 [8.1] | 3 [6.8] | 3 [10] |  |
| *neutral* | 3 [4.1] | 1 [2.3] | 2 [6.7] |  |
| *Useful* | 24 [32.4] | 10 [22.7] | 14 [46.7] |  |
| *Very useful* | 37 [50] | 29 [65.1] | 8 [26.7] |  |
| **Q14f: *Rewards/ collecting points*** |  |  |  |  |
|  |  |  |  |  |
|  |  |  |  |  |
| *Not useful* | 16 [21.6] | 9 [20.4] | 7 [23.3] |  |
| *Less useful* | 12 [16.2] | 6 [13.6] | 6 [20] |  |
| *neutral* | 16 [21.6] | 9 [20.4] | 7 [23.3] |  |
| *Useful* | 14 [18.9] | 8 [18.2] | 6 [20] |  |
| *Very useful* | 16 [21.6] | 12 [27.3] | 4 [13.3] |  |
| **Q14g:** ***Individual feedback by a real person (based on data)*** |  |  |  |  |
|  |  |  |  |  |
|  |  |  |  |  |
| *Not useful* | 2 [2.7] | 2 [4.5] | 0 [0] |  |
| *Less useful* | 1 [1.4] | 1 [2.3] | 0 [0] |  |
| *neutral* | 17 [23] | 6 [13.6] | 11 [36.7] |  |
| *Useful* | 21 [28.4] | 8 [18.2] | 13 [43.3] |  |
| *Very useful* | 33 [44.6] | 27 [61.4] | 6 [20] |  |
| **Q14h: *Individual feedback of a virtual agent (based on data)*** |  |  |  |  |
|  |  |  |  |  |
|  |  |  |  |  |
| *Not useful* | 12 [16.2] | 5 [11.4] | 7 [23.3] |  |
| *Less useful* | 16 [21.6] | 8 [18.2] | 8 [26.7] |  |
| *neutral* | 19 [25.7] | 11 [25] | 8 [26.7] |  |
| *Useful* | 13 [17.6] | 11[ [25] | 2 [6.7] |  |
| *Very useful* | 14 [18.9] | 9 [20.4] | 5 [16.7] |  |
| **Q14i: *Learning/ education section*** |  |  |  |  |
|  |  |  |  |  |
|  |  |  |  |  |
| *Not useful* | 5 [6.8] | 4 [9.1] | 1 [3.3] |  |
| *Less useful* | 4 [5.4] | 3 [6.8] | 1 [3.3] |  |
| *neutral* | 22 [29.7] | 13 [29.5] | 9 [30] |  |
| *Useful* | 18 [24.3] | 9 [20.4] | 9 [30] |  |
| *Very useful* | 25 [33.8] | 15 [34.1] | 10 [33.3] |  |
| **Q14j: *Share progress with family and friends*** |  |  |  |  |
|  |  |  |  |  |
|  |  |  |  |  |
| *Not useful* | 26 [35.1] | 20 [45.5] | 6 [20] |  |
| *Less useful* | 7 [9.5] | 5 [11.4] | 2 [6.7] |  |
| *neutral* | 13 [17.6] | 7 [15.9] | 6 [20] |  |
| *Useful* | 14 [18.9] | 5 [11.4] | 9 [30] |  |
| *Very useful* | 14 [1839] | 7 [15.9] | 7 [23.3] |  |
| **Q14k: *A bulletin board with best scores*** |  |  |  |  |
|  |  |  |  |  |
|  |  |  |  |  |
| *No support* | 26 [35.1] | 15 [34.1] | 11 [36.7] |  |
| *less support* | 13 [17.6] | 9 [20.4] | 4 [13.3] |  |
| *neutral/ unsure* | 10 [13.5] | 2 [4.5] | 8 [26.7] |  |
| *some support* | 13 [17.6] | 8 [18.2] | 5 [16.7] |  |
| *Strong support* | 12 [16.2] | 10 [22.7] | 2 [6.7] |  |
| **Q15a:** *"How important would it be to you to have the following data stored in the system and available for you to view?"* ***Current medication*** |  |  |  |  |
|  |  |  |  |  |
|  |  |  |  |  |
| *Not important* | 12 [16.2] | 8 [18.2] | 4 [13.3] |  |
| *Less important* | 5 [6.8] | 3 [6.8] | 2 [6.7] |  |
| *Neutral/ unsure* | 9 [12.2] | 5 [11.4] | 4 [13.3] |  |
| *Important* | 15 [20.3] | 8 [18.2] | 7 [23.3] |  |
| *Very important* | 33 [44.6] | 20 [45.5] | 13 [43.3] |  |
| **Q15b: *Laboratory results*** |  |  |  |  |
|  |  |  |  |  |
|  |  |  |  |  |
| *Not important* | 6 [8.1] | 3 [6.8] | 3 [10] |  |
| *Less important* | 2 [2.7] | 1 [2.3] | 1 [3.3] |  |
| *Neutral/ unsure* | 8 [10.8] | 4 [9.1] | 4 [13.3] |  |
| *Important* | 15 [20.3] | 8 [18.2] | 7 [23.3] |  |
| *Very important* | 43 [58.1] | 28 [63.6] | 15 [50] |  |
| **Q15c: *Diagnosis*** |  |  |  |  |
|  |  |  |  |  |
|  |  |  |  |  |
| *Not important* | 6 [8.1] | 3 [6.8] | 3 [10] |  |
| *Less important* | 0 [0] | 0 [0] | 0 [0] |  |
| *Neutral/ unsure* | 8 [10.8] | 4 [9.1] | 4 [13.3] |  |
| *Important* | 17 [23] | 9 [20.5] | 8 [26.7] |  |
| *Very important* | 43 [58.1] | 28 [63.6] | 15 [50] |  |
| **Q15d: *Overall risk score*** |  |  |  |  |
|  |  |  |  |  |
|  |  |  |  |  |
| *Not important* | 5 [6.8] | 2 [4.5] | 3 [10] |  |
| *Less important* | 1 [1.4] | 1 [2.3] | 0 [0] |  |
| *Neutral/ unsure* | 4 [5.4] | 3 [6.8] | 1 [3.3] |  |
| *Important* | 22 [29.7] | 13 [29.5] | 9 [30] |  |
| *Very important* | 42 [56.8] | 25 [56.8] | 17 [56.7] |  |
| **Q16a:** *"What is your opinion on the following, possible features?"* **Daily step count** |  |  |  |  |
|  |  |  |  |  |
|  |  |  |  |  |
| *Do not like it/ not needed* | 5 [6.8] | 3 [6.8] | 2 [6.7] |  |
| *Not necessarily needed* | 4 [5.4] | 2 [4.5] | 2 [6.7] |  |
| *Neutral/ unsure* | 19 [25.7] | 11 [25] | 8 [26.7] |  |
| *I like it* | 19 [25.7] | 8 [18.2] | 11 [36.7] |  |
| *I like it very much/ should be implemented* | 27 [36.5] | 20 [45.5] | 7 [23.3] |  |
| **Q16b: *Updates on recommended training pulse*** |  |  |  |  |
|  |  |  |  |  |
|  |  |  |  |  |
| *Do not like it/ not needed* | 3 [4.1] | 2 [4.5] | 1 [3.3] |  |
| *Not necessarily needed* | 1 1.4] | 1 [2.3] | 0 [0] |  |
| *Neutral/ unsure* | 10 [13.5] | 5 [11.4] | 5 [16.7] |  |
| *I like it* | 23 [31.1] | 11 [25] | 12 [40] |  |
| *I like it very much/ should be implemented* | 37 [50] | 25 [56.8] | 12 [40] |  |
| **Q16c: *Training progress/ evaluation*** |  |  |  |  |
|  |  |  |  |  |
|  |  |  |  |  |
| *Do not like it/ not needed* | 5 [6.8] | 2 [4.5] | 3 [10] |  |
| *Not necessarily needed* | 1 [1.4] | 1 [2.3] | 0 [0] |  |
| *Neutral/ unsure* | 12 [16.2] | 9 [20.5] | 3 [10] |  |
| *I like it* | 24 [32.4] | 12 [27.3] | 12 [40] |  |
| *I like it very much/ should be implemented* | 32 [43.2] | 20 [45.5] | 12 [40] |  |
| **Q16d: *Advice on healthy diet*** |  |  |  |  |
|  |  |  |  |  |
|  |  |  |  |  |
| *Do not like it/ not needed* | 7 [9.5] | 6 [13.6] | 1 [3.3] |  |
| *Not necessarily needed* | 6 [8.1] | 4 [9.1] | 2 [6.7] |  |
| *Neutral/ unsure* | 16 [21.6] | 12 [27.3] | 4 [13.3] |  |
| *I like it* | 17 [23] | 6 [13.6] | 11 [36.7] |  |
| *I like it very much/ should be implemented* | 28 [37.8] | 16 [36.4] | 12 [40] |  |
| **Q16e: *Evaluation of sleep quality*** |  |  |  |  |
|  |  |  |  |  |
|  |  |  |  |  |
| *Do not like it/ not needed* | 13 [17.6] | 7 [15.9] | 6 [20] |  |
| *Not necessarily needed* | 4 [5.4] | 3 [6.8] | 1 [3.3] |  |
| *Neutral/ unsure* | 9 [12.2] | 6 [13.6] | 3 [10] |  |
| *I like it* | 19 [25.7] | 10 [22.7] | 9 [30] |  |
| *I like it very much/ should be implemented* | 29 [39.2] | 18 [40.9] | 11 [36.7] |  |
| **Q16f: *Tool for stress management*** |  |  |  |  |
|  |  |  |  |  |
|  |  |  |  |  |
| *Do not like it/ not needed* | 13 [17.6] | 9 [20.5] | 4 [13.3] |  |
| *Not necessarily needed* | 3 [4.1] | 3 [6.8] | 0 [0] |  |
| *Neutral/ unsure* | 12 [16.2] | 9 [20.5] | 3 [10] |  |
| *I like it* | 23 [31.1] | 10 [22.7] | 13 [43.3] |  |
| *I like it very much/ should be implemented* | 23 [31.1] | 13 [29.5] | 10 [33.3] |  |
| **Q16g: ECG measurements during exercise** |  |  |  |  |
|  |  |  |  |  |
|  |  |  |  |  |
| *Do not like it/ not needed* | 5 [6.8] | 4 [9.1] | 1 [3.3] |  |
| *Not necessarily needed* | 6 [8.1] | 4 [9.1] | 2 [6.7] |  |
| *Neutral/ unsure* | 9 [12.2] | 3 [6.8] | 6 [20] |  |
| *I like it* | 19 [25.7] | 6 [13.6] | 13 [43.3] |  |
| *I like it very much/ should be implemented* | 35 [47.3] | 27 [61.4] | 8 [26.7] |  |
| **Q16h: *Documentation of blood pressure*** |  |  |  |  |
|  |  |  |  |  |
|  |  |  |  |  |
| *Do not like it/ not needed* | 5 [6.8] | 3 [6.8] | 2 [6.7] |  |
| *Not necessarily needed* | 3 [4.1] | 2 [4.5] | 1 [3.3] |  |
| *Neutral/ unsure* | 7 [9.5] | 4 [9.1] | 3 [10] |  |
| *I like it* | 23 [21.1] | 9 [20.5] | 14 [46.7] |  |
| *I like it very much/ should be implemented* | 36 [48.6] | 26 [59.1] | 10 [33.3] |  |
| **Q16i: *Documentation of blood oxygen saturation*** |  |  |  |  |
|  |  |  |  |  |
|  |  |  |  |  |
| *Do not like it/ not needed* | 11 [14.9] | 5 [11.4] | 6 [20] |  |
| *Not necessarily needed* | 6 [8.1] | 4 [9.1] | 2 [6.7] |  |
| *Neutral/ unsure* | 5 [6.8] | 4 [9.1] | 1 [3.3] |  |
| *I like it* | 22 [29.7] | 11 [25] | 11 [36.7] |  |
| *I like it very much/ should be implemented* | 30 [40.5] | 20 [45.5] | 10 [33.3] |  |
| **Q16j: *Display of weekly exercise volume*** |  |  |  |  |
|  |  |  |  |  |
|  |  |  |  |  |
| *Do not like it/ not needed* | 1 [1.4] | 1 [2.3] | 0 [0] |  |
| *Not necessarily needed* | 5 [6.8] | 3 [6.8] | 2 [6.7] |  |
| *Neutral/ unsure* | 11 [14.9] | 8 [18.2] | 3 [10] |  |
| *I like it* | 25 [33.8] | 15 [34.1] | 10 [33.3] |  |
| *I like it very much/ should be implemented* | 32 [43.2] | 17 [38.6] | 15 [50] |  |
| **Q17:** *"Would you like to be able to see the data in 'real time' or would daily summaries sufficient?"* |  |  |  |  |
|  |  |  |  |  |
|  |  |  |  |  |
| *Daily summaries* | 42 [56.8] | 33 [75] | 9 [30] |  |
| *Real time* | 21 [28.4] | 6 [13.6] | 15 [50] |  |
| *Do not know/ undecided* | 11 [14.9] | 5 [11.4] | 6 [20]5 |  |
| **Q18:** *"If the system would send out messages/ push notifications: How often would you want to receive messages and reminders??"* |  |  |  |  |
|  |  |  |  |  |
|  |  |  |  |  |
| *Multiple times a day* | 4 [5.4] | 2 [4.5] | 2 [6.7] |  |
| *Once a day* | 20 [27] | 12 [27.3] | 8 [26.7] |  |
| *Every other day* | 19 [25.7] | 10 [22.7] | 9 [30] |  |
| *Never* | 7 [9.5] | 4 [9.1] | 3 [10] |  |
| *Prefer a flexible schedule* | 24 [32.4] | 16 [36.4] | 8 [26.7] |  |
| **Q19:** *"Would you want to communicate with the system; be able to respond/ react to messages or start communication?"* |  |  |  |  |
|  |  |  |  |  |
|  |  |  |  |  |
| *No* | 17 [23] | 12 [27.3] | 5 [16.7] |  |
| *Yes* | 24 [32.4] | 14 [31.8] | 10 [33.3] |  |
| *I do not know/ undecided* | 12 [16.2] | 4 [9.1] | 8 [26.7] |  |
| *Depends on the ability of the system* | 21 [28.4] | 14 [31.8] | 7 [23.3] |  |

Data is shown as n [%]. § Only patients answering “yes” in Q1 were asked. *Multiple answers possible; ^#^ some participants refrained from answering as they were non-smokers/ had no opinion.
